# CT image-based biomarkers acquired by AI-based algorithms for the opportunistic prediction of falls

**DOI:** 10.1259/bjro.20230014

**Published:** 2023-05-16

**Authors:** Daniel Liu, Neil C Binkley, Alberto Perez, John W Garrett, Ryan Zea, Ronald M Summers, Perry J Pickhardt

**Affiliations:** 1 Department of Radiology, University of Wisconsin School of Medicine & Public Health, Madison, WI, USA; 2 Osteoporosis Clinical Research Program, University of Wisconsin School of Medicine & Public Health, Madison, WI, USA; 3 Imaging Biomarkers and Computer-Aided Diagnosis Laboratory, Department of Radiology and Imaging Sciences, National Institutes of Health Clinical Center, Bethesda, MD, USA

## Abstract

**Objective:**

Evaluate whether biomarkers measured by automated artificial intelligence (AI)-based algorithms are suggestive of future fall risk.

**Methods:**

In this retrospective age- and sex-matched case–control study, 9029 total patients underwent initial abdominal CT for a variety of indications over a 20-year interval at one institution. 3535 case patients (mean age at initial CT, 66.5 ± 9.6 years; 63.4% female) who went on to fall (mean interval to fall, 6.5 years) and 5494 controls (mean age at initial CT, 66.7 ± 9.8 years; 63.4% females; mean follow-up interval, 6.6 years) were included. Falls were identified by electronic health record review. Validated and fully automated quantitative CT algorithms for skeletal muscle, adipose tissue, and trabecular bone attenuation at the level of L1 were applied to all scans. Uni- and multivariate assessment included hazard ratios (HRs) and area under the receiver operating characteristic (AUROC) curve.

**Results:**

Fall HRs (with 95% CI) for low muscle Hounsfield unit, high total adipose area, and low bone Hounsfield unit were 1.82 (1.65–2.00), 1.31 (1.19–1.44) and 1.91 (1.74–2.11), respectively, and the 10-year AUROC values for predicting falls were 0.619, 0.556, and 0.639, respectively. Combining all these CT biomarkers further improved the predictive value, including 10-year AUROC of 0.657.

**Conclusion:**

Automated abdominal CT-based opportunistic measures of muscle, fat, and bone offer a novel approach to risk stratification for future falls, potentially by identifying patients with osteosarcopenic obesity.

**Advances in knowledge:**

There are few well-established clinical tools to predict falls. We use novel AI-based body composition algorithms to leverage incidental CT data to help determine a patient’s future fall risk.

## Introduction

Older adults are at elevated risk for falling with subsequent serious morbidity and mortality. Falls are the most common cause of injuries in older adults, and the incidence is increasing.^1^ Overall, 25% of males and 37% of females aged 65 years and above have a reported fall once within 12 months, with the highest incidence for geriatric inpatients.^2^ The etiology of falls is often multifactorial; risk factors include both extrinsic phenomena (including loose carpets, phone cords, etc.) and intrinsic factors (including sarcopenia, medication use, psychological impairment, and sensory deficits).^3,4^ Early identification of patients at risk of falling is paramount to reduce morbidity and mortality, as interventions exist that can be pre-emptively implemented to prevent falls.

The American Geriatrics Society recommends annual falls and instability screening in people 65 years of age and older. However, there is currently no gold-standard approach to estimate fall risk despite over 100 fall risk assessment tools published.^5^ Conventional methods include geriatrician gestalt and risk assessment tools such as the Timed Up and Go test or the St. Thomas Risk Assessment Tool in Falling Elderly Inpatients.^6–8^ However, the reported efficacy of these approaches is variable. Analysis by an interdisciplinary geriatric care team was found to have the best performance, with a sensitivity of 56% and a specificity of 80%.^8^ Induced regression models had similar performance.^8^ Another study found the sensitivity of fall risk assessment tools, such as the Morse Fall Scale or the Hendrich II Fall Risk Model, ranged from 57 to 100%, while the specificity ranged from 25 to 69%.^9^ Further, if an observational approach to estimate fall risk is utilized, interrater reliability can vary depending on the skill of physicians or other members of the care team. A recent study by Wilbur et al found only moderate agreement between geriatricians’ predictions of fallers and non-fallers.^5^ Moreover, sarcopenia and sarcopenic obesity should predispose to falling but implementation of these concepts into clinical care has been hampered by lack of easily applicable consensus definitions. Widespread availability of objective imaging-based measures could potentially improve this gap in clinical care.

Opportunistic imaging offers a novel approach to evaluation of fall risk. Clinical imaging, particularly CT, is often performed for a variety of targeted indications but in addition contains valuable biometric information that can potentially be utilized to provide insight to a patient’s health status.^10^ While the association of CT-based measures and falls is not well documented, some CT-based measures have been shown to provide insight into a patient’s functional status. For example, using CT imaging, it was found that increased intramuscular adipose tissue in the proximal hip muscles, particularly the hip abductors, was associated with increased gait variability and poorer balance.^11^ As such, we aim to determine the clinical value obtained from previously developed automated AI-based tools. We hypothesized that opportunistic CT measures of muscle, fat and bone could help predict future falls.

The purposes of this study were to determine: (1) if differences exist in fully automated CT-based measures of bone, muscle, and total adipose tissue (TAT) between patients who went on to fall and healthy controls, (2) which biomarkers or combinations of biomarkers best predict the risk of future fall, and (3) thresholds for these measures that provide relatively high specificity.

## Methods

### Patient cohort

This HIPAA-compliant investigation was approved by the institutional review board at the University of Wisconsin School of Medicine and Public Health and the Office of Human Subjects Research Protection at the National Institutes of Health Clinical Center. Patients who underwent abdominal CT for a variety of indications over a 20-year interval at the University of Wisconsin were eligible for inclusion. Excluded were patients without clinical follow-up from initial CT to either a fall (cases) or last contact date (controls), or failure of the automated CT tools. Specifically, a total of 12, 59, and 41 cases were excluded for failure of the muscle, bone, and fat tools to generate a result, each representing <1% of cases. A detailed search script of the electronic health record was used to identify coded fall outcomes for all patients occurring after their CT scan.

As a result, 9029 total patients were included in the final analysis, with 3535 patients who went on to fall and 5494 sex- and age-matched controls (utilizing a control–case ratio between 1.5 and 2). This patient group was a separate cohort from the one used to train and test the automated algorithms, and there was no additional training or learning involved. By intention, the clinical indication for imaging and the abdominal CT technique both varied widely in this heterogeneous patient population, to test the generalizability of this approach.

### Automated CT imaging biomarker

Algorithms to automatically segment and quantify the CT-based biomarkers used in this study were previously developed, trained, and tested; success rates, normative values, and changes over time for these measures were established in separate publications.^12–20^ Succinctly, the original DICOM abdominal CT images for each scan were all automatically and uniformly reconstructed to a 3 mm slice thickness and CT numbers normalized across vendors. Next, a deep learning spinal segmentation and labeling algorithm identified each vertebral level from T12 to L5,^21^ followed by level-specific measures of skeletal muscle (L1), abdominal TAT (L1), and trabecular bone (L1). While the tools assess all organ systems in a given abdominal CT image slice, these specific measures were chosen because obesity and sarcopenia are well-documented intrinsic factors that increase the risk of falls.^22,23^ While bone mineral density (BMD) was not directly associated with increased fall risk, it has been shown to be indicative of sarcopenia and therefore was included in the analysis to investigate its use as a secondary biomarker.^24^

To measure trabecular bone density, an automated region of interest (ROI) at the L1 vertebral trabecular space was designed to simulate the well-established manual approach, generating the median attenuation value [in Hounsfield unit (HU)] within the ROI.^25,26^ This ROI was placed using a deep learning model with a modified U-Net architecture^27^ trained on a large, diverse patient population (14,290 unique patients were represented in the training dataset).^20^ At each slice in the volume, the deep learning placed a ROI and the median CT number (HU) was measured. The three contiguous cephalad and caudad images to the index single-slice L1 level were included, and the lowest of the corresponding HU values from these seven slices was used to increase the sensitivity for osteoporosis.^20^

A deep learning muscle algorithm segmented the skeletal muscles at the level of L1 and L3, including the paraspinal, psoas, and body wall musculature. This segmentation used a U-Net model architecture^28^ trained on a patient cohort of 51 unique patients.^17^ Although both cross-sectional area for assessing myopenia and mean attenuation values for myosteatosis were both derived, we have found that the latter was generally the more valuable imaging biomarker.^29^

Finally, visceral and subcutaneous fat compartments were segmented by an algorithm consisting of five steps: body masking, noise reduction, adipose tissue labeling, visceral and subcutaneous adipose tissue (VAT and SAT) separation (combining as TAT), and quantification.^16^ This semantic segmentation classifies all voxels in the selected slices as either visceral fat, subcutaneous fat, or neither; this is achieved using fuzzy c-means to cluster fatty tissues and active contour models to separate subcutaneous and visceral fat.^30^ From this segmented image, the areas of SAT, VAT, and TAT are calculated (in cm^2^). In addition, the ratio of VAT to SAT areas (VSR) is calculated. Cross-sectional areas were calculated at each vertebral level, although the analysis was focused on the L1 level.

**Figure 1.**
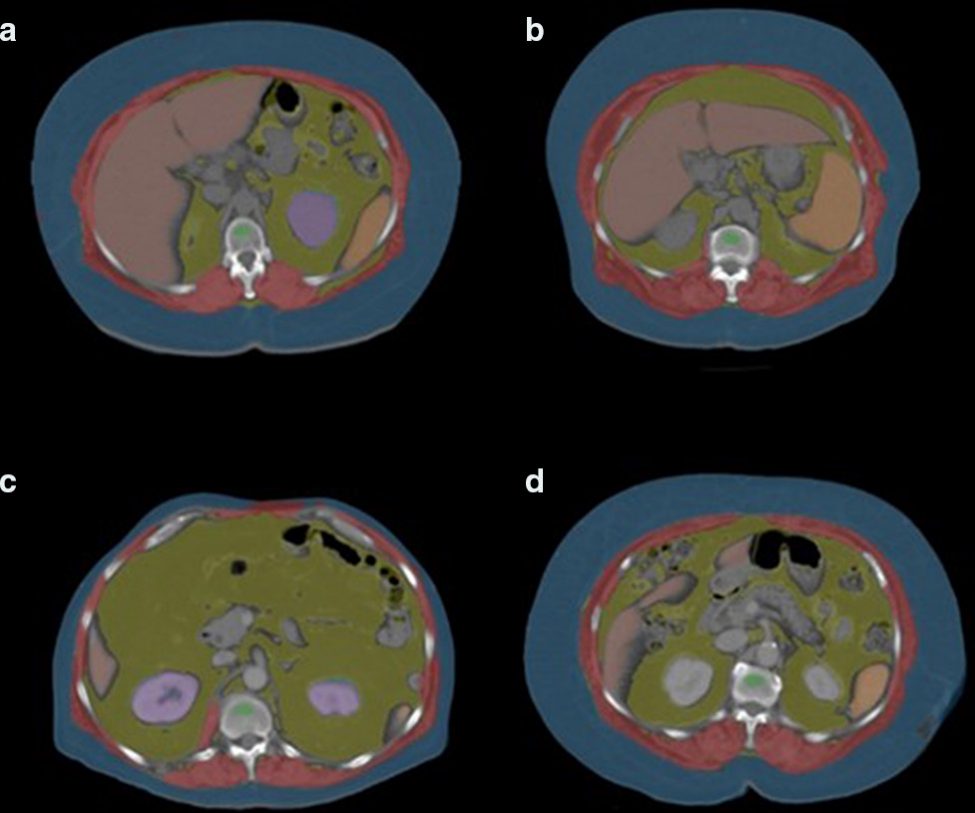
Non-contrast transverse (axial) CT images at the L1-level superimposed with AI-based fully automated tissue segmentation in four fall cases with abnormal muscle, bone, and fat measures relative to the 80% specificity thresholds (Table 4). (a) 58-year-old female, abdominal CT for urolithiasis. Presented 19 years later with a fall after her legs gave out while standing and multiple rib fractures. Abnormal L1-level measures for TAT (659.2 cm^2^), muscle attenuation (−14.1 HU), and trabecular attenuation (107.6 HU). (b) 69-year-old female, CT for recurrent UTIs. Presented 12 years later with a fall after slipping on a wet surface and distal radius fracture. Abnormal L1-level measures for TAT (648.5 cm^2^), muscle attenuation (−28.5 HU), and trabecular attenuation (92.0 HU). (c) 67-year-old male, CT after fall revealed multiple vertebral compression fractures. Abnormal L1-level measures for TAT (627.4 cm^2^), muscle attenuation (−11.5 HU), and trabecular attenuation (103.9 HU). (d) 63-year-old female, CT urography for hematuria. Presented 10 years later with a fall after tripping on stairs and multiple rib fractures. Abnormal L1-level measures for TAT (710.6 cm^2^), muscle attenuation (−23.8 HU), and trabecular attenuation (98.2 HU). For reference, the red overlay = skeletal muscle, yellow = visceral adipose tissue, blue = subcutaneous adipose tissue, and green = a region of interest in the L1 vertebral body trabecular bone. HU, Hounsfield unit; TAT, total adipose tissue.

All the CT-based segmentations were visually overlayed on the original CT images (Figure 1). This provided quality assurance for individual cases, as we could confirm the algorithm was correctly segmenting the intended structures. Technical failure rates for these tools were observed to be around 1% for the current patient cohort which is consistent with previously documented failure rates on the order of 2–3% or less in large heterogeneous cohorts.^31^

### Statistical analysis

Summary statistics were compiled for patients with and without falls. *p*-values were derived using two-sided *t*-tests for variables with normal distribution and the Wilcoxon rank sum test for variables where the assumption of a normal distribution did not hold. Uni- and multivariate Cox proportional hazard models were used to derive hazard ratios (HRs) with 95% confidence intervals (CIs), comparing both the highest and lowest risk quartiles, as well the highest risk to the other three quartiles. Uni- and multivariate area under the receiver operating characteristic (ROC) area under curve (AUC) analyses were performed using the DeLong method. For ROC curve analysis, defined time intervals included 2, 5, and 10 years. Logistic regression and event-free survival analysis were used to compute separate thresholds for achieving specificity levels of both 80 and 90% at 10 years. The time-to-event plots were also visually assessed for differences between measures. R (v. 3.6, R Project for Statistical Computing) was used for statistical analyses.

## Results

### Patient characteristics

The final study cohort consisted of 9029 patients, with a mean age at initial CT of 66.6 ± 9.7 years and including 5728 (63.4%) women. 3535 case patients (mean age at initial CT, 66.5 ± 9.6 years; 63.4% female) went on to have a fall (mean time from CT to fall, 6.5 years), whereas 5494 controls (mean age at initial CT, 66.7 ± 9.8 years; 63.4% females) did not go on to fall as of their most recent clinical follow-up (mean follow-up interval, 6.6 years)

### Automated CT-based measures and diagnostic performance for falls

**Figure 2.**
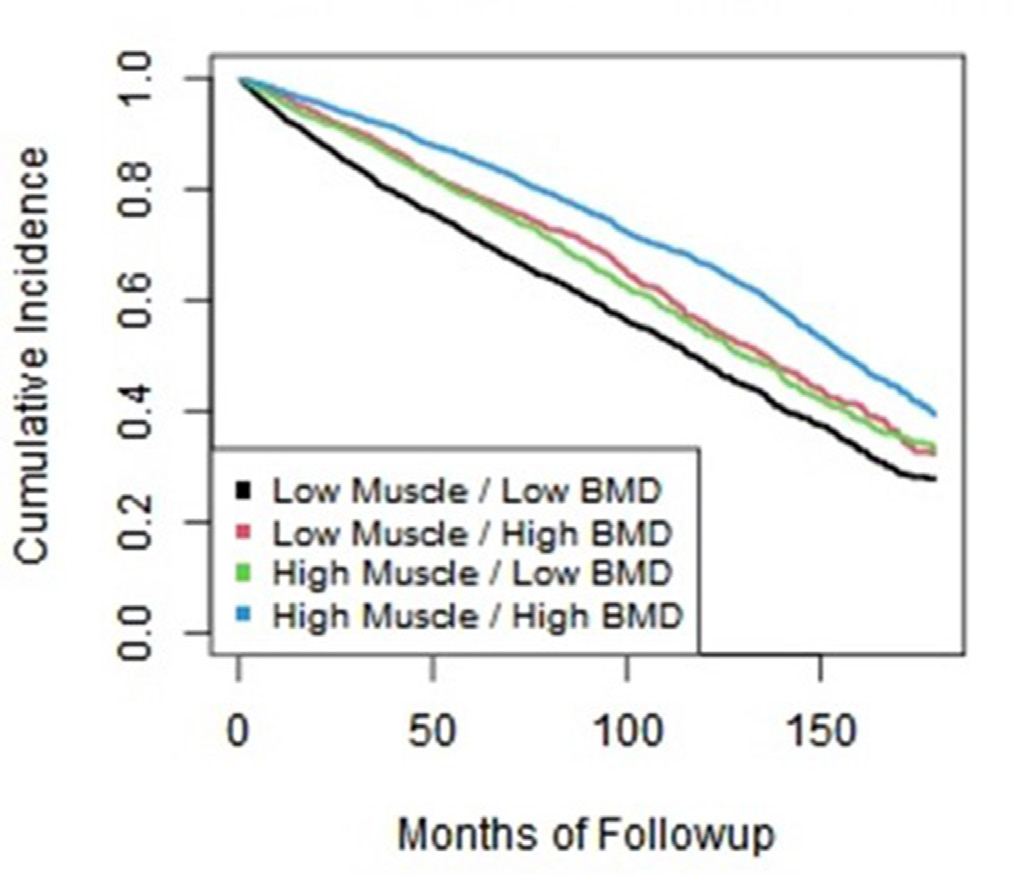
Kaplan–Meier time-to-event plots for falls. Kaplan–Meier plot of falls over time when combining L1 muscle and bone HU according to the high or low values relative to median. Complementary value for muscle and bone assessment is apparent. BMD, bone mineral density; HU, Hounsfield unit.

Low skeletal muscle attenuation values (muscle HU), high TAT area, and low trabecular bone attenuation values (BMD) were all associated with a significantly increased risk of fall. Summary statistics for these CT-based body composition measures are shown in Table 1. Falls HRs (with 95% CI) comparing the highest to lowest risk quartiles were 1.82 (1.65–2.00) for low muscle HU, 1.31 (1.19–1.44) for high TAT area, and 1.91 (1.74–2.11) for low BMD. Of note, the fall HR for low muscle area using the highest *vs* lowest methodology was 1.02 (0.95–1.10). Falls HRs (with 95% CI) comparing the highest risk quartile to the other three quartiles were 1.48 (1.37–1.60) for low L1 muscle HU, 1.18 (1.01–1.27) for high TAT area, and 1.52 (1.40–1.64) for low L1 BMD. Of note, the fall HR for low muscle area using the highest *vs* all three methodology was also 1.02 (0.95–1.10). The Kaplan–Meier plot for L1 muscle HU and bone HU values by quartile is shown in Figure 2.

**Table 1.** Summary statistics

Automated CT biomarker		No fall (*n* = 5494)	Fall (*n* = 3535)
L1 Muscle HU	Mean (SD)	20.0 (17.6)	20.5 (17.0)
	Median (IQR)	21.3 (8.4–32.6)	17.4 (9.2–32.3)
L1 muscle area (cm^2^)	Mean (SD)	113.7 (35.6)	115.0 (36.8)
	Median (IQR)	109.4 (87.5–135.2)	109.4 (88.8–136.2)
L1 TAT area (cm^2^)	Mean (SD)	285.5 (166.5)	301.0 (169.2)
	Median (IQR)	263.9 (158.4–384.3)	277.1 (175.5–399.2)
L1 trabecular HU	Mean (SD)	130.9 (40.1)	133.0 (38.0)
	Median (IQR)	129.6 (104.7–153.8)	132.0 (107.1–155.5)

HU, Hounsfield unit; IQR = interquartile range; SD = standard deviation; TAT = total adipose tissue.

**Figure 3.**
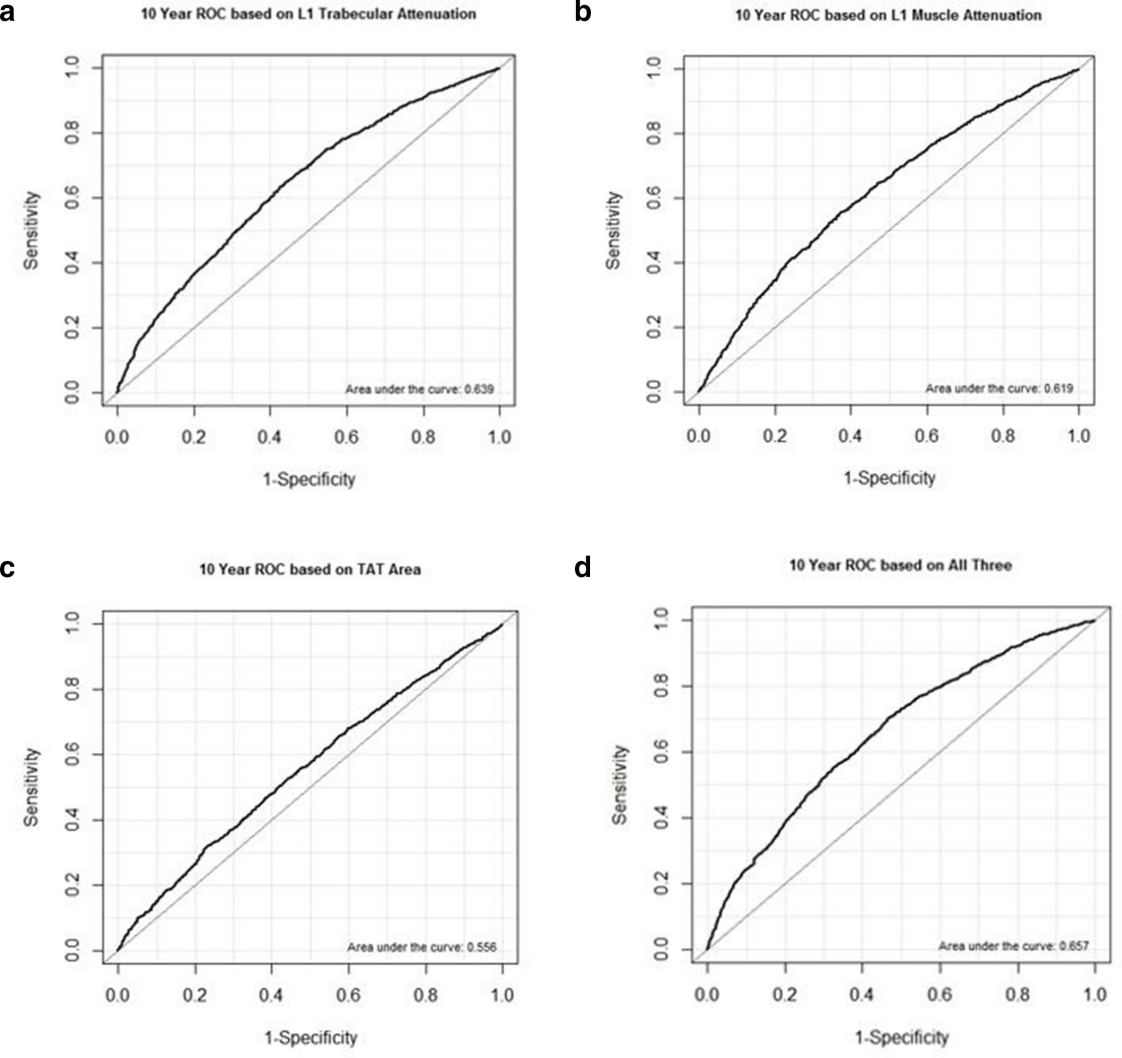
10-year ROC curves. 10-year ROC curves for predicting falls using automated CT-based L1 trabecular attenuation (A, AUC = 0.639), L1 muscle attenuation (B, AUC = 0.619), and L1 TAT area (C, AUC = 0.556). Multivariate combinations of these three CT biomarkers improved the overall predictive value (D, AUC = 0.657). AUC, area under the curve; ROC, receiver operating characteristic; TAT, total adipose tissue.

The 2-, 5-, and 10-year ROC AUC values for predicting falls according to univariate CT-based biomarkers are shown in Table 2. For example, the 10-year ROC AUC values for L1 muscle HU, TAT area, and BMD were 0.619, 0.556, and 0.639, respectively (Figure 3). For males only, the 10-year ROC AUC values for L1 muscle HU, TAT area, and BMD were 0.636, 0.554, and 0.629, respectively. For females only, the 10-year ROC AUC values for L1 muscle HU, TAT area, and BMD were 0.607, 0.563, and 0.647, respectively. Multivariate combinations of these CT biomarkers improved the predictive value according to ROC AUC, as shown in Table 3. Notably, the 10-year AUROC from combining muscle and TAT was 0.622, while combining muscle, and TAT, and bone was 0.657 (Figure 3).

**Table 2.** Univariate ROC AUC values for CT biomarkers

		5-year ROC AUC	10-year ROC AUC
Automated CT biomarker	HR (95% CI)	(*n* = 4570)	(*n* = 4205)
		(Fall = 1571)	(Fall = 2960)
L1 muscle HU	1.82 (1.65–2.00)	0.606	0.619
L1 muscle area (cm^2^)	1.02 (0.95–1.10)	0.500	0.509
L1 TAT area (cm^2^)	1.31 (1.19–1.44)	0.543	0.556
L1 trabecular HU	1.91 (1.74–2.11)	0.616	0.639

AUC, area under the curve; CI, confidence interval; HR, hazard ratio; HU, Hounsfield unit; ROC, receiver operating characteristic; TAT = total adipose tissue.

**Table 3.** Multivariate ROC AUC values for CT biomarkers

	2-year ROC AUC	5-year ROC AUC	10-year ROC AUC
Automated CT biomarker	(*n* = 4802)	(*n* = 4570)	(*n* = 4205)
	(Falls = 703)	(Falls = 1571)	(Falls = 2960)
Bone, muscle, fat	0.651	0.639	0.657
Muscle and fat	0.619	0.610	0.622
Bone and muscle	0.648	0.637	0.656

AUC, area under the curve; Bone = L1 trabecular HU; Fat = L1 TAT Area (cm^2^); Muscle = L1 Muscle HU;ROC, receiver operating characteristic; TAT, total adipose tissue.

Table 4 depicts CT biomarker thresholds for achieving 80 and 90% levels of specificity for falls at 10 years. For example, using an L1 muscle HU threshold of 12.65 HU provides an 80% specificity with a sensitivity of 34.1%, using a TAT area threshold of 392.5 cm^2^ provides an 80% specificity with a sensitivity of 26.3%, and using a L1 trabecular attenuation of 114.5 HU provides an 80% specificity with a sensitivity of 36.3%. The CT biomarker thresholds used to maintain 90% specificity generally resulted in very low sensitivities for future falls.

**Table 4.** CT biomarker thresholds to achieve target specificity

Automated CT biomarker	Threshold	Specificity	Sensitivity
L1 trabecular HU	114.5 HU	80%	36.3%
	99.9 HU	90%	22.5%
L1 Muscle HU	12.65 HU	80%	34.1%
	4.05 HU	90%	19.0%
L1 TAT area (cm^2^)	392.5 cm^2^	80%	26.3%
	477.7 cm^2^	90%	14.9%

HU, Hounsfield unit; TAT, total adipose tissue.

## Discussion

In this retrospective age- and sex-matched case–control study, fully automated measurements of skeletal muscle attenuation, TAT area, and vertebral trabecular attenuation (BMD) from abdominal CT scans in patients who went on to fall were evaluated and compared with controls who did not have a fall event to determine if they had clinical value. All CT images were acquired prior to the fall and obtained for other clinical indications. We found that patients who went on to fall had significantly lower skeletal muscle attenuation values, higher TAT area, and lower vertebral trabecular attenuation on their initial CT scan compared with age-matched controls. Based on univariate regression analysis, these automated measures were significantly associated with the risk of future falls. Multivariate combinations of these factors improved the predictive value. Patient sex had limited effect on the predictive value. To limit the number of false-positive results in the “do no harm” spirit of opportunistic screening, optimal univariate thresholds for falls at 10 years with a specificity of 80% were determined. Those values for L1 muscle attenuation, TAT, and L1 trabecular attenuation were 12.7 HU, 392.5 cm^2^, and 114.5 HU, respectively. Automated abdominal CT-based measures of muscle, fat, and bone provide useful risk stratification for future falls.

There are no established gold-standard metrics for falls. However, a number of physiologic changes confer an increased risk for falls, including sarcopenia, obesity, and a combination termed sarcopenic obesity.^22,23,32^ Sarcopenic obesity patients were found to have reduced physical performance, greater risk of frailty, and at a higher risk of adverse musculoskeletal outcomes compared to patients with sarcopenia, obesity, or neither condition.^33,34^ Patients with sarcopenic obesity had a higher falls risk compared to controls, with a risk ratio of 1.30 (95% CI 1.10–1.54), and a higher falls risk compared to obesity alone, with a risk ratio of 1.17 (95% CI 1.01–1.36).^32^ The fall risk in sarcopenic obesity patients was similar to patients with sarcopenia alone.^32^

Since muscle strength is not necessarily correlated with muscle mass, some have proposed the terms dynapenia (low muscle strength) and sarcopenia (low muscle mass).^35–37^ Interestingly, Scott et al found that when separating sarcopenia and dynapenia, dynapenic obesity and not sarcopenic obesity was predictive of increased fall risk in middle-aged and older adults.^38^ Our results more closely reflect this finding. Low muscle CT attenuation but not low muscle mass was predictive of future falls (Table 2). Low muscle CT attenuation is thought to be suggestive of dynapenia. Myosteatosis is closely related to dynapenia, and since adipose tissue and skeletal muscle have a different attenuation on CT, this difference can be detected.^39^ Likewise, muscle density and not size correlates well with muscle strength and can easily be measured by CT scan.^40^ This study shows low muscle attenuation was consistently among the best CT-based measures for predicting falls, which corresponds well with the established literature correlating muscle weakness and falls.

The relationship between low BMD and falls is unclear. To our knowledge, there is no current established relationship between the two in the literature, although low BMD is known to increase the risk of complications, like hip fractures. The best uni- and multivariate predictors involved muscle and bone measurements. It is possible that there is a confounding variable in the relationship. Some evidence suggests sarcopenia may be associated with low BMD. Confirmed sarcopenic patients were found to have significantly lower BMD at multiple sites, including the lumbar spine and hip.^24^ This could explain why low BMD was found to be predictive of falls. On the other hand, it is possible there is an unestablished relationship between osteoporotic patients and fall risk that was uncovered by the algorithm. More research would be needed to investigate such a relationship.

Risk factors for falls are heterogeneous and include both intrinsic and extrinsic causes. Thus, it is not surprising that the predictive value of this method is limited. The predictive value could potentially be improved with inclusion of additional CT biomarkers to detect other intrinsic causes of falls, including cognitive impairment, arthritis, and visual deficits.^3^ Some of these physiologic changes could certainly be captured with CT imaging, as biomarkers like liver attenuation or aortic calcification likewise provide insight into a patient’s overall health.^41,42^ Although not all extrinsic components to fall risk will be captured by CT imaging, with a specificity of 80%, these measures can outperform previously established algorithms for evaluating fall risk. Further, there is value in determining a patient’s risk based on intrinsic components, as these patients can be identified as those needing further workup (*e.g.* fully looking into both the intrinsic and extrinsic risks) with the currently established and more time-consuming approaches.

We acknowledge limitations to this study. This retrospective cohort was from a single institution with a relatively homogeneous racial population consisting of roughly 90% Caucasian. External validation of these automated body composition tools in a more diverse cohort may be necessary in other practice settings to confirm generalizability. This should involve more diverse patient cohorts and inherent variations in CT scanners and technique. In addition, the possibility exists that some patient falls were not documented or missed by the EHR search. The exact mechanism of fall and sequelae are not always documented, limiting the analysis for the cohort. The CTC protocol consists of unenhanced imaging. A previous study noted the effect of contrast enhancement on bone and muscle attenuation.^43^ Therefore, corrections may be needed to extrapolate these results to account for the impact of i.v. contrast. Finally, while the use of CT-based muscle measurement at the L1 level has recently been validated, the L3 level may be preferable to L1 for abdominal adipose tissue measurement.^29^

In conclusion, CT-based body composition measurements were compared in patients who had a fall event and controls who did not have a fall event. Low muscle attenuation, high subcutaneous adipose tissue, and low trabecular bone attenuation were the strongest independent risk factor for future falls. Low muscle attenuation, representing weakness, may be the best overall predictor in the context of the currently known pathophysiology for falling. Combining these measurements improved the overall predictive value. Optimal thresholds for each metric were identified, and below these values, further fall risk assessment may be warranted. Use of L1-level measures means these results could be applied to chest CT which typically includes the level of L1. Evaluation of fall risk is challenging, but use of CT-based measurements offers a novel approach for opportunistic prediction of future falls.

## References

[1] Berková M, Berka Z . Falls: a significant cause of morbidity and mortality in elderly people. Vnitr Lek 2018; 64: 1076–83. 30606025

[2] von Renteln-Kruse W, Krause T, Dieckmann P, Vogel J . Geriatric patients’ mobility status as reflected by the relevant items of the BARTHEL index and in-hospital falls. J Am Geriatr Soc 2006; 54: 1012–13. doi: 10.1111/j.1532-5415.2006.00755.x 16776807

[3] Perell KL, Nelson A, Goldman RL, Luther SL, Prieto-Lewis N, Rubenstein LZ . Fall risk assessment measures: an analytic review. J Gerontol A Biol Sci Med Sci 2001; 56: M761–6. doi: 10.1093/gerona/56.12.m761 11723150

[4] Oliver D, Daly F, Martin FC, McMurdo MET . Risk factors and risk assessment tools for falls in hospital in-patients: a systematic review. Age Ageing 2004; 33: 122–30. doi: 10.1093/ageing/afh017 14960426

[5] Wilbur J, Jogerst G, Butler N, Xu Y . How accurate are geriatricians’ fall predictions? BMC Geriatr 2022; 22: 436. doi: 10.1186/s12877-022-03129-w 35585524 PMC9118876

[6] Podsiadlo D, Richardson S . The timed”up & go”: a test of basic functional mobilitv for frail elderly persons. J Am Geriatr Soc 1991; 39: 142–48. doi: 10.1111/j.1532-5415.1991.tb01616.x 1991946

[7] Oliver D, Britton M, Seed P, Martin FC, Hopper AH . Development and evaluation of evidence based risk assessment tool (stratify) to predict which elderly inpatients will fall: case-control and cohort studies. BMJ 1997; 315: 1049–53. doi: 10.1136/bmj.315.7115.1049 9366729 PMC2127684

[8] Marschollek M, Rehwald A, Wolf K-H, Gietzelt M, Nemitz G, zu Schwabedissen HM, et al . Sensors vs. experts - a performance comparison of sensor-based fall risk assessment vs. conventional assessment in a sample of geriatric patients. BMC Med Inform Decis Mak 2011; 11: 48. doi: 10.1186/1472-6947-11-48 21711504 PMC3141375

[9] Chapman J, Bachand D, Hyrkäs K . Testing the sensitivity, specificity and feasibility of four falls risk assessment tools in a clinical setting. J Nurs Manag 2011; 19: 133–42. doi: 10.1111/j.1365-2834.2010.01218.x 21223413

[10] Pickhardt PJ . Value-added opportunistic CT screening: state of the art. Radiology 2022; 303: 241–54. doi: 10.1148/radiol.211561 35289661 PMC9083232

[11] Addison O, Young P, Inacio M, Bair W-N, Prettyman MG, Beamer BA, et al . Hip but not thigh intramuscular adipose tissue is associated with poor balance and increased temporal gait variability in older adults. Curr Aging Sci 2014; 7: 137–43. doi: 10.2174/1874609807666140706150924 24998419 PMC4480674

[12] Jang S, Graffy PM, Ziemlewicz TJ, Lee SJ, Summers RM, Pickhardt PJ . Opportunistic osteoporosis screening at routine abdominal and thoracic ct: normative l1 trabecular attenuation values in more than 20 000 adults. Radiology 2019; 291: 360–67. doi: 10.1148/radiol.2019181648 30912719 PMC6492986

[13] Pickhardt PJ, Lee SJ, Liu J, Yao J, Lay N, Graffy PM, et al . Population-based opportunistic osteoporosis screening: validation of a fully automated ct tool for assessing longitudinal bmd changes. Br J Radiol 2019; 92: 20180726. doi: 10.1259/bjr.20180726 30433815 PMC6404831

[14] Summers RM, Baecher N, Yao J, Liu J, Pickhardt PJ, Choi JR, et al . Feasibility of simultaneous computed tomographic colonography and fully automated bone mineral densitometry in a single examination. J Comput Assist Tomogr 2011; 35: 212–16. doi: 10.1097/RCT.0b013e3182032537 21412092 PMC3077119

[15] Graffy PM, Liu J, Pickhardt PJ, Burns JE, Yao J, Summers RM . Deep learning-based muscle segmentation and quantification at abdominal CT: application to a longitudinal adult screening cohort for sarcopenia assessment. BJR 2019; 92: 20190327. doi: 10.1259/bjr.20190327 31199670 PMC6724622

[16] Lee SJ, Liu J, Yao J, Kanarek A, Summers RM, Pickhardt PJ . Fully automated segmentation and quantification of visceral and subcutaneous fat at abdominal CT: application to a longitudinal adult screening cohort. BJR 2018; 91: 20170968. doi: 10.1259/bjr.20170968 29557216 PMC6223139

[17] Burns JE, Yao J, Chalhoub D, Chen JJ, Summers RM . A machine learning algorithm to estimate sarcopenia on abdominal CT. Acad Radiol 2020; 27: 311–20. doi: 10.1016/j.acra.2019.03.011 31126808

[18] Sandfort V, Yan K, Pickhardt PJ, Summers RM . Data augmentation using generative adversarial networks (cyclegan) to improve generalizability in CT segmentation tasks. Sci Rep 2019; 9: 16884. doi: 10.1038/s41598-019-52737-x 31729403 PMC6858365

[19] Yao JH et al . A multi-center milestone study of clinical vertebral CT segmentation. Comput Med Imaging Graph 2016; 49: 16–28. 26878138 10.1016/j.compmedimag.2015.12.006 PMC5527557

[20] Pickhardt PJ, Nguyen T, Perez AA, Graffy PM, Jang S, Summers RM, et al . Improved CT-based osteoporosis assessment with a fully automated deep learning tool. Radiol Artif Intell 2022; 4(5): e220042. doi: 10.1148/ryai.220042 36204542 PMC9530763

[21] Yan K, Lu L, Summers RM . Unsupervised body part regression via spatially self-ordering convolutional neural networks. 2018 IEEE 15th International Symposium on Biomedical Imaging (ISBI 2018); Washington, DC; 2018. pp. 1022–25. doi: 10.1109/ISBI.2018.8363745

[22] Yeung SSY, Reijnierse EM, Pham VK, Trappenburg MC, Lim WK, Meskers CGM, et al . Sarcopenia and its association with falls and fractures in older adults: A systematic review and meta-analysis. J Cachexia Sarcopenia Muscle 2019; 10: 485–500. doi: 10.1002/jcsm.12411 30993881 PMC6596401

[23] G R Neri S, S Oliveira J, B Dario A, M Lima R, Tiedemann A, Newman A . Does obesity increase the risk and severity of falls in people aged 60 years and older? A systematic review and meta-analysis of observational studies. J Gerontol A Biol Sci Med Sci 2020; 75: 952–60. doi: 10.1093/gerona/glz272 31750880

[24] Scott D, Johansson J, McMillan LB, Ebeling PR, Nordstrom P, Nordstrom A . Associations of sarcopenia and its components with bone structure and incident falls in Swedish older adults. Calcif Tissue Int 2019; 105: 26–36. doi: 10.1007/s00223-019-00540-1 30899995

[25] Pickhardt PJ, Pooler BD, Lauder T, del Rio AM, Bruce RJ, Binkley N . Opportunistic screening for osteoporosis using abdominal computed tomography scans obtained for other indications. Ann Intern Med 2013; 158: 588–95. doi: 10.7326/0003-4819-158-8-201304160-00003 23588747 PMC3736840

[26] Pickhardt PJ, Lee LJ, del Rio AM, Lauder T, Bruce RJ, Summers RM, et al . Simultaneous screening for osteoporosis at CT colonography: bone mineral density assessment using MDCT attenuation techniques compared with the DXA reference standard. J Bone Miner Res 2011; 26: 2194–2203. doi: 10.1002/jbmr.428 21590738 PMC3304444

[27] Vladimir Iglovikov AS . TernausNet: U-Net with VGG11 Encoder Pre-Trained on ImageNet for Image Segmentation. ArXiv e-prints; 2018.

[28] Ronneberger O, Fischer P, Brox T . U-net: convolutional networks for biomedical image segmentation. Med Image Comput Comput Assist Interv 2015; 9351: 234–41. doi: 10.1007/978-3-319-24574-4

[29] Pickhardt PJ, Perez AA, Garrett JW, Graffy PM, Zea R, Summers RM . Fully automated deep learning tool for sarcopenia assessment on ct: l1 versus l3 vertebral level muscle measurements for opportunistic prediction of adverse clinical outcomes. AJR Am J Roentgenol 2022; 218: 124–31. doi: 10.2214/AJR.21.26486 34406056 PMC9028606

[30] Yao J, Weaver JB, Molthen RC, Sussman DL, Summers RM . Fully automated adipose tissue measurement on abdominal CT. In: Weaver JB, Molthen RC , eds. SPIE Medical Imaging; Lake Buena Vista, Florida. Bellingham; 3 March 2011. doi: 10.1117/12.878063

[31] Pooler BD, Garrett JW, Southard AM, Summers RM, Pickhardt PJ . Technical adequacy of fully automated artificial intelligence body composition tools: assessment in a heterogeneous sample of external CT examinations. American Journal of Roentgenology 2023. doi: 10.2214/AJR.22.28745 37095663

[32] Gandham A, Mesinovic J, Jansons P, Zengin A, Bonham MP, Ebeling PR, et al . Falls, fractures, and areal bone mineral density in older adults with sarcopenic obesity: A systematic review and meta-analysis. Obes Rev 2021; 22: e13187. doi: 10.1111/obr.13187 33491333

[33] Batsis JA, Villareal DT . Sarcopenic obesity in older adults: aetiology, epidemiology and treatment strategies. Nat Rev Endocrinol 2018; 14: 513–37. doi: 10.1038/s41574-018-0062-9 30065268 PMC6241236

[34] Yazar T, Olgun Yazar H . Prevalance of sarcopenia according to decade. Clin Nutr ESPEN 2019; 29: 137–41. doi: 10.1016/j.clnesp.2018.11.005 30661677

[35] Hughes VA, Frontera WR, Wood M, Evans WJ, Dallal GE, Roubenoff R, et al . Longitudinal muscle strength changes in older adults: influence of muscle mass, physical activity, and health. J Gerontol A Biol Sci Med Sci 2001; 56: B209–17. doi: 10.1093/gerona/56.5.b209 11320101

[36] Goodpaster BH, Park SW, Harris TB, Kritchevsky SB, Nevitt M, Schwartz AV, et al . The loss of skeletal muscle strength, mass, and quality in older adults: the health, aging and body composition study. J Gerontol A Biol Sci Med Sci 2006; 61: 1059–64. doi: 10.1093/gerona/61.10.1059 17077199

[37] Clark BC, Manini TM . Sarcopenia =/= dynapenia. J Gerontol A Biol Sci Med Sci 2008; 63: 829–34. doi: 10.1093/gerona/63.8.829 18772470

[38] Scott D, Sanders KM, Aitken D, Hayes A, Ebeling PR, Jones G . Sarcopenic obesity and dynapenic obesity: 5-year associations with falls risk in middle-aged and older adults. Obesity (Silver Spring) 2014; 22: 1568–74. doi: 10.1002/oby.20734 24585708

[39] Sugiyama Y, Ishizu Y, Ando Y, Yokoyama S, Yamamoto K, Ito T, et al . Obesity and myosteatosis: the two characteristics of dynapenia in patients with cirrhosis. Eur J Gastroenterol Hepatol 2021; 33: e916–21. doi: 10.1097/MEG.0000000000002303 35048658

[40] Wang L, Yin L, Zhao Y, Su Y, Sun W, Chen S, et al . Muscle density, but not size, correlates well with muscle strength and physical performance. J Am Med Dir Assoc 2021; 22: 751–59. doi: 10.1016/j.jamda.2020.06.052 32768372

[41] Graffy PM, Sandfort V, Summers RM, Pickhardt PJ . Automated liver fat quantification at nonenhanced abdominal ct for population-based steatosis assessment. Radiology 2019; 293: 334–42. doi: 10.1148/radiol.2019190512 31526254 PMC6822771

[42] Pickhardt PJ, Graffy PM, Zea R, Lee SJ, Liu J, Sandfort V, et al . Automated ct biomarkers for opportunistic prediction of future cardiovascular events and mortality in an asymptomatic screening population: a retrospective cohort study. Lancet Digit Health 2020; 2: e192–200. doi: 10.1016/S2589-7500(20)30025-X 32864598 PMC7454161

[43] Perez AA, Pickhardt PJ, Elton DC, Sandfort V, Summers RM . Fully automated CT imaging biomarkers of bone, muscle, and fat: correcting for the effect of intravenous contrast. Abdom Radiol (NY) 2021; 46: 1229–35. doi: 10.1007/s00261-020-02755-5 32948910

